# Generating Buoyancy in a Sea of Uncertainty: Teachers Creativity and Well-Being During the COVID-19 Pandemic

**DOI:** 10.3389/fpsyg.2020.614774

**Published:** 2021-01-18

**Authors:** Ross C. Anderson, Tracy Bousselot, Jen Katz-Buoincontro, Jandee Todd

**Affiliations:** ^1^Inflexion, Eugene, OR, United States; ^2^School of Education, Drexel University, Philadelphia, PA, United States

**Keywords:** creative self-efficacy, creative growth mindset, creative anxiety, secondary traumatic stress, teacher well-being, teacher buoyancy

## Abstract

The global coronavirus disease 2019 (COVID-19) pandemic has caused significant uncertainty for students and teachers. During this time, teacher and student creative beliefs and affect play a supportive role in adaptively managing stress, finding joy, and bouncing back from inevitable setbacks with resilience. Developing an adaptive orientation to creativity is a critically important step in helping teachers deal with the challenges and stress of reaching their students through distance learning, especially the most marginalized. This study aims to understand how teacher creativity linked to well-being in the face of COVID-19-related school shutdowns and how teachers planned to adapt creatively to distance learning through the guidance of a summer creative teaching training institute. Results from this sequential mixed method study demonstrated important relationships. Creative self-efficacy in teaching related to teacher buoyancy in the face of setbacks. Creative growth mindset related to teachers’ general positive affect in teaching. Lowered creative anxiety related to reduced effects of secondary traumatic stress and general negative affect in teaching. Environmental support and encouragement for creativity in schools may be foundational for teacher well-being by enhancing teachers’ dispositional joy, general positive affect, and reducing general negative affect. Results suggested additional stress and loss of creativity for most teachers due to the COVID-19 pandemic alongside substantial capacity for creative adaptations with the support of training for creativity in teaching and learning.

## Introduction

The coronavirus disease 2019 (COVID-19) pandemic has disrupted school communities across the globe. Students and teachers have experienced high amounts of stress and mental health challenges (Jones, 2020). During this time of uncertainty, teachers have been forced to pivot and change their instruction and curriculum to distance learning and connect with students virtually. As a result, the demands for teacher resilience, creativity, stress management, and tolerance for ambiguity and anxiety are high. Due to the continuing changes from the pandemic, researchers struggle to document how teachers cope with these surmounting stressors and how they develop their capacity to teaching for creativity. Little research has investigated this issue, especially in schools serving high proportions of students marginalized due to socioeconomic factors, who often struggle to participate in distance learning. In the face of this global crisis, teachers’ creative beliefs and affect may play a critical role in facing uncertainty, managing stress, and maintaining overall well-being as a teacher.

This mixed method study addresses this gap by examining teachers’ experience in the United States working through the first months of the COVID-19 school shutdown and how they developed strategies to teach for creativity during the spring and summer of the 2019–2020 school year. We investigate the link between educators’ well-being and the creative beliefs and affect that underlie their approach to teaching for creativity. The results of this study hope to shed light on the teacher experience during the COVID-19 pandemic, the link between their well-being and creative beliefs and affect, and the creative adaptations for the classroom that resulted from engagement in professional learning for creativity in the classroom.

### The Stressful State of Teaching

Many educators choose to enter the teaching profession in order to make a difference in students’ lives. To that newly aspiring educator, the profession may provide the promise of connection, creativity, fulfilment, and joy in cultivating a love of learning in their students. And, yet, even before the additional stressors of the COVID-19 pandemic, the teaching profession in the United States has been suffering from widespread teacher dissatisfaction and disengagement. Educators have been on strike across the United States for the past few years. Remarkable declines in enrollment in US teacher preparation programs has left leaders bemoaning the shortage of teachers in almost every US state, especially in rural areas (Sutcher et al., 2016; García and Weiss, 2019). Even before the pandemic, education funding was barely back to, or still lagging behind, pre-recession 2008 levels in many places—the pandemic has greatly exacerbated financial challenges (Strauss, 2020). Underlining the new crisis of educator disengagement and stress, a recent Gallup poll found nearly half of US teachers are actively looking for a different job or watching for opportunities to change professions (Gallup, 2014). For many would-be and existing educators, stressful circumstances suppress the joy, and creativity of the profession.

Unfortunately, the current level of teacher disengagement affects the quality of students’ learning experiences, especially in critical areas like relationship building. In a 2017 Educator Quality of Work Life Survey (American Federation of Teachers, 2017), 61% of educators reported their work was “always” or “often” stressful—a rate that is likely higher for teachers serving in schools with high concentrations of student poverty. Socioeconomically segregated schools struggle with high turnover, initiative overload, and *secondary traumatic stress* for educators—the stress experienced by caring for students experiencing trauma (Walker, 2019). Only recently has the education field shed light on this especially powerful form of stress that can arise from the caregiving role many teachers provide their students. Originally conceptualized within the social work field, secondary traumatic stress represents the behaviors and emotions that result from knowing about traumatizing events experienced by another individual for whom you care for or want to help (Bride et al., 2004). For teachers, this type of stress is likely exacerbated by the pandemic.

The school environment continues to become more stressful for teachers, with few, if any, efforts aimed at alleviating this issue directly. The experience of high stress and negative affect can diminish teachers’ creative resources—their creative beliefs, affect, thinking, and behaviors (Anderson, 2020). Creativity is a critical part of sustaining the joy of work: In fact, on the whole, recent college graduates care more about being creative in their job than getting a high salary (National Association of Colleges and Employers, 2014). The fundamental yearning for creativity in people relates to a need for agency and autonomy in both personal and professional life (Bandura, 2006). These needs are no less powerful for educators than other professionals, and they are likely key conditions for joy in the classroom. Simply put, teachers, today, especially during the crisis of a global pandemic, seek support and permission to be agentic and creative in their work, which may play a role in mitigating stress and enhancing well-being during crises. The links between teachers’ creative beliefs and affect, the support for creativity in their school, their overall well-being, and their capacity to adapt to challenging circumstances are the focus for this current study.

### Teacher Creative Development and Well-Being in Schools

Few, if any, studies have investigated the relationship between teachers’ beliefs and affect toward creativity, their perceptions of support for creativity in their environment, and their overall well-being in their work, especially during the current global pandemic. Across studies from different international contexts, teachers’ beliefs about creativity clearly play a role in their enacted classroom practices (Bereczki and Kárpáti, 2018). Moreover, according to research from various international contexts, the pedagogical environment designed and facilitated by the teacher plays a role in the creative development of students (Davies et al., 2013). Teachers’ beliefs and affect matter in their enactment of creative practices in the classroom, and that enactment matters in the creative development of students.

Different aspects of an individuals’ creative resources have been linked to overall well-being across the lifespan (Cohen, 1989) and the resilience to bounce back from setbacks in the face of adversity (Sweetman et al., 2011). For instance, Maslow (1962) proposed individuals’ creative resources facilitated self-actualization and full integration of the person. Regarding well-being in the workplace, studies covering more than 12,000 daily diary entries describe how positive emotions and motivations nurture creative performance, the importance of meaningful work to everyday well-being, and how being creative in work leads to the richest emotional experiences (Amabile, 2017). Not surprisingly, research highlights the important role the work environment plays in teachers’ creative development and well-being.

Organizations that foster positive psychological and emotional engagement at work also support creative performance across a range of professional settings (Amabile et al., 1996; Sweetman et al., 2011). And, yet, classroom observation research across two decades illustrates how most school environments remain deprived of conditions that foster opportunities for creative growth (Katz-Buonincontro and Anderson, 2018; Pitts et al., 2018). An organizational climate that supports creativity likely plays a role in teachers’ overall well-being in their work, including their experience of positive and negative affect, their state of joy, the stress they carry to care for their students, and their buoyancy, or resilience to everyday setbacks. This study provides an initial test of the link between support for creativity in school and teacher well-being in their work during the COVID-19 pandemic.

### Unlocking Teachers’ Creative Growth for Well-Being

Certain creative beliefs and affect likely play a role in shaping teacher well-being during times of great uncertainty and stress. An individual’s creative action results, in part, from holding a creative growth mindset and approaching failures as opportunities (Amabile et al., 1996; Hass et al., 2016) and from feeling self-efficacious and agentic about one’s potential to be creative (Karwowski and Beghetto, 2018). Emphasizing openness to mistakes and improvement, a creative growth mindset likely relates to teachers’ attitude and affect when facing uncertainty. Creative growth is only possible if individuals are open to vulnerability and are willing to take risks, especially when facing uncertainty—a central component to the creative process (Amabile, 2017). In fact, intellectual risk-taking moderates the effect of creative confidence on actual creative behavior and achievement across different domains—if an individual is unwilling to take intellectual risks, the link between creative confidence and behavior disappears (Beghetto et al., 2020). Growth also depends on educators’ work being personally meaningful and fulfilling, but high levels of stress and lack of encouragement make creative risk-taking unlikely (Beghetto, 2019). Taking risks in the face of great challenges, such as distance learning, requires confidence. Creative self-efficacy in teaching should lead to greater ease and persistence through hindrances and challenges (Ventura et al., 2015) and should link to enhanced resilience and buoyancy in the face of setbacks. This study provides an initial test of these hypotheses.

To produce creativity often requires persisting through ambiguous challenges and tinkering with possibilities where none existed before. To create something new when uncertainty is high requires tolerance for ambiguity, where a lower tolerance may prematurely close teachers off to new connections or possibilities (Lubart et al., 2013). Conceptually intertwined, creative anxiety can result from facing uncertainty in teaching, which could contribute to higher levels of negative affect and stress. Creative anxiety is the unease, worry, and dread that arises from having to think in an open-ended and creative way, focus on novelty, or come up with a unique way of doing something (Daker et al., 2019). To reduce creative anxiety and enhance teachers’ creative beliefs through periods of stress may relate to the environmental support around them and their access to related training experiences. Professional development experiences can focus on supporting creative development, buoyancy, well-being, and enduring joy by focusing on the moment-to-moment openings for creativity in classroom routines that shape relationships and the environment for learning (Gajda et al., 2017). These moments can cement teachers’ beliefs, affect, and attitudes toward creativity; they could also build resilience and well-being, reducing stress, anxiety, and negative affect in teachers’ work.

Importantly, positive emotions are one part of creative growth; it is unlikely that holding more positive beliefs and attitudes about creativity will necessarily mean less-negative affective experiences in teaching, especially during a pandemic. For instance, teachers experiencing professional development to learn how to integrate drama-based exercises into their teaching reported the full spectrum of emotions from fear, vulnerability, and nervousness to joy, thrill, and excitement (Anderson and Beard, 2018). Their growth required passing through initial stress and fear. Still, a sense of safety was critical to their willingness and capacity to be playful in teaching and learning and build confidence through risk-taking, even when fear was present. A common human response to uncertainty is fear and anxiety and, in fact, negative moods can enhance alertness in the creative process (De Dreu et al., 2008). If teachers feel high levels of anxiety facing new challenges that demand creativity during a school shutdown, it is also likely they could take on greater secondary traumatic stress due to difficulty in managing the uncertainty that their students face, as well. In this way, their own creative anxiety and the trauma their students face could become cumulative stress. In contrast, if teachers can learn to manage creative anxiety, perhaps they can also reduce the intensity of secondary traumatic stress. This study includes an initial test of this relationship.

When teachers experience difficult emotional states in professional development demanding creativity, they may become more alert to the regular opportunities that offer scaffolded creative risk-taking for students, even in the midst of a crisis. This study explores the relationships between teachers’ creative beliefs and affect, perceived support for creativity, and their overall well-being in teaching to shed light on some of these potential links.

### Present Study

To understand how teachers’ creative resources support their adaptability and buoyancy in the face of a crisis, such as the COVID-19 pandemic, it is helpful to know how their adaptive and maladaptive creative beliefs and affect relate to their overall well-being in teaching during a crisis of unprecedented magnitude and uncertainty. Moreover, it is important to know more about the stress they experience, their approach to dealing with that stress, and the creative adaptations they invent to engage their students’ creativity in the difficulty of distance learning. These insights can help shape new directions for future research and support for teacher development. The following research questions guided the present study.

1. During the COVID-19 school shutdown in the spring of 2020, how do creative beliefs and affect and environmental support for creativity relate to factors of well-being, including joy, general positive and negative affect in teaching, secondary traumatic stress, and buoyancy in teaching?
2. Among the creativity factors that relate to common aspects of well-being, which demonstrate the strongest relationship?
3. According to teachers, how did the COVID-19 pandemic affect their capacity to provide creative opportunities to students?
4. According to teachers, how did the COVID-19 pandemic cause more stress than usual as a teacher and what did teachers do to reduce that stress?
5. With more than 30 h of professional learning experience focused on creative teaching and learning techniques, what creative ideas did teachers generate to support the social-emotional and pedagogical challenges they and their students would face in the 2020–2021 school year?

## Materials and Methods

This explanatory sequential mixed method design (Creswell and Plano Clark, 2018) balanced descriptive statistics, bivariate correlations, and results from several multiple regressions alongside thematic analysis of teachers’ written reflections and ideas for distance learning during the COVID-19 pandemic. Phase 1 quantitative survey data aimed to understand the links between different psychological well-being and creative beliefs and affect experienced by teachers during the COVID-19 pandemic in the Spring of 2020 using close-ended survey items to address research questions 1 and 2 and open-ended survey responses to address research questions 3 and 4. Phase 2 data included teachers’ written plans drawn from their concluding submissions to the 15 h asynchronous online Foundation Course and a 14 h synchronous virtual Summer Institute. The ideas generated represent teachers’ adaptations of creative teaching and learning strategies in a distance learning format. Qualitative thematic analysis of the written plans addressed research question 5, aiming to understand teachers’ approach to deal with the stress and uncertainty of the COVID-19 pandemic. The results include the perspectives of a sample (*N* = 57) K–12 teachers from both rural and urban settings.

### Sample

The sample of participating teachers in this study enrolled in a blended arts integration for creative engagement professional development experience based on broad dissemination efforts to dozens of schools and districts across Oregon and California that were eligible to participate, based on federal eligibility requirements (i.e., at least 20% of families with school-aged children in the district lived in poverty). The recruitment efforts took place during the April–June 2020 period when schools were shut down due to the COVID-19 pandemic. As such, the sample of teachers who selected to participate were likely already interested and invested in creativity and the arts. The sample of *n* = 57 teachers included *n* = 4 teachers identifying as Hispanic, *n* = 3 teachers identifying as multiple race and ethnicities, and the majority, *n* = 50 teachers identifying as white. Teachers predominantly identified as females (*n* = 45 or 79%) with one teacher preferring not to identify their gender. Teachers came from *n* = 32 schools, so, generally, they participated on their own or with a couple of colleagues at their school. In one school, *n* = 8 teachers selected to enroll and participate. These schools represented mostly rural regions that ranged in size and extent of remoteness and diversity of socioeconomic factors, such as race, ethnicity, and economic privilege. Teachers had an average of 14.68 years of teaching experience, ranging from 1 to 45 years in the classroom. Teachers represented all levels of K–12 education and content area specialization.

### Procedures

#### Teacher Professional Development

The Foundation Course for Creative Engagement and the virtual Summer Institute provided teachers a research-based understanding of creativity in teaching and learning through reflective, experiential, and arts integrated instruction and application. Teachers learned and applied a variety of teaching techniques to integrate creative and artistic processes into their instruction and curriculum, starting with brief creative routines. Participating teachers consented to participate in all research activities and agreed to complete the online course material and attend the Summer Institute in order to receive payment for their time.

#### Online Learning Materials

**FIGURE 1:**
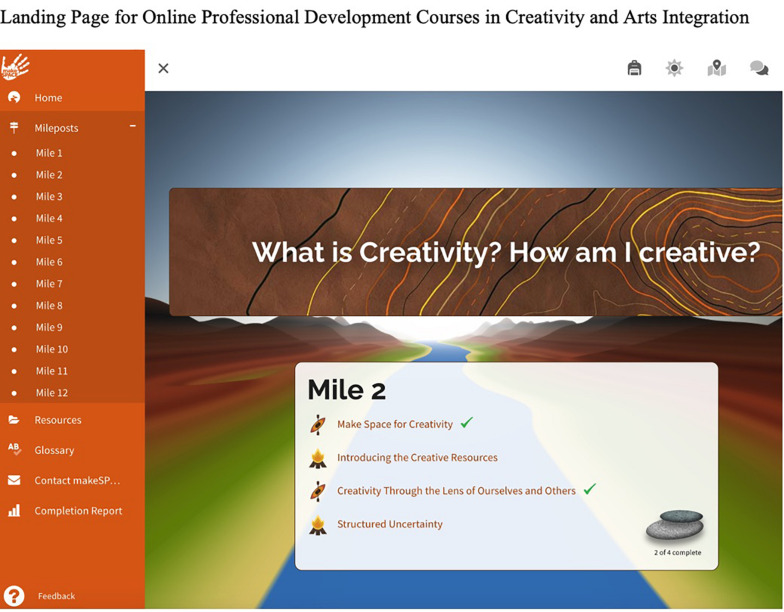
Landing page for online professional development courses in creativity and arts integration.

**FIGURE 2:**
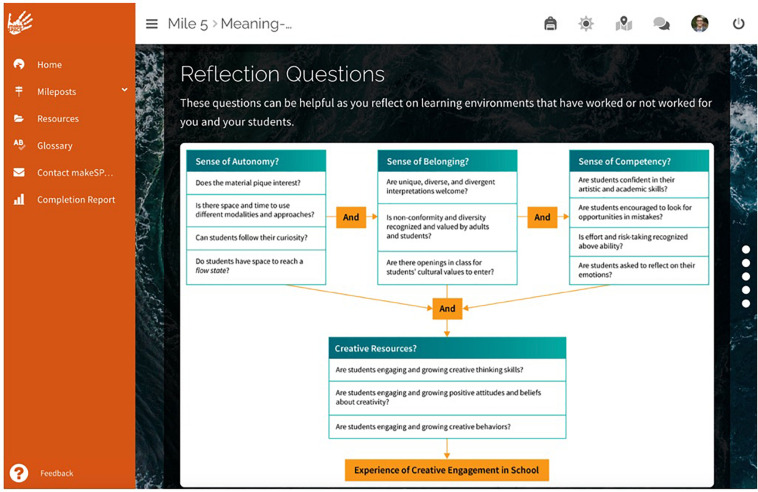
Sample course page from foundation course.

The online Foundation Course (depicted in Figures 1, 2) was made up of 12 modules with 1–9 lessons per module. Modules included interactive instructional packages with video, narrated slideshows, pop-up interactives, creative exercises, reflective processes, and brief creative assignments. All content was designed, written, and narrated by professional instructional designers with expertise in creativity in education and arts integration. Teachers logged into the online platform and completed the pre-course survey with open- and close-ended items prior to starting the course. Project partners sent each participant a sketch journal and a small pack of *metaphor cards* to use in the course, where each card has a clip art image of a common object or scene. Table 1 describes the focus of the modules and lessons; teachers were required to complete each module to proceed. The course was designed to support teachers with useful mental models, language, examples, and routines for exploring the creative process in teaching and learning. For instance, teachers explored their own personal creative resources (Anderson, 2020)—creative attitudes, creative thinking, and creative behaviors, responding to the question—*How am I creative?—*and creating a metaphorical *creative avatar* collage in their journal (see Supplementary Appendix for examples). They photographed their work, uploaded it to the course, and shared it with colleagues and facilitators.

**TABLE 1:** Scope and sequence for make SPACE foundation course for creative engagement in arts integration—*the river journey* (metaphor used across the course).

**Welcome and orientation**	
Mile 1	Lessons: makeSPACE for creativity; Introducing the creative resources
**What is creativity? How am I creative?**	
Mile 2	Lessons: Creativity through the lends of ourselves and others; Stories of creative risk-taking and growth with arts integration in the classroom; Reflecting on the development of personal creative resources
Mile 3	Lessons: Teachers as artists of pedagogy
Mile 4	Lessons: How are you creative? How are you creative? Creative resources as teaching tools; Making your creative avatar
**How do I makeSPACE for creativity?**	
Mile 5	Lessons: Conditions for creative engagement; Flow stories; Meaning-making through creative engagement; Patterns; Cultivating conditions and planning for creative engagement
Mile 6	Lessons: Creative routines; Routines and intentions; Why creative routines? Vocabulary gesture reflection; Many uses game and reflection; 10 min routines; Implementation idea; Choose a routine
**What is arts integration?**	
Mile 7	Lessons: Role of artistic practice; Skills and sensibilities; Art is a verb! Learning through the arts; Treasure hunt; Still life
Mile 8	Lessons: What is arts integration? Tools for integration
**How do I begin to integrate?**	
Mile 9	Lessons: Arts integration: How? Refining intentions and review; Designing for quality arts integration; When you integrate the arts…; Core practices; Share your avatar; Which routine did you practice?
Mile 10	Lessons: Metaphorical thinking; Metaphor hunting; A metaphor for the self; Metaphor gestures and homework
Mile 11	Lessons: Reflective practices; Why reflection? Your selfie; Reflective routines; Metaphor card reflection; Opportunities to reflect; Notice…; Share your reflections
**Final stretch**	
Mile 12	Lessons: Braided channel; Portage; Entering the delta; Share the experience you designed; The take-out; Congratulations

The course content summarized the state of research in education, motivation, creativity, and the arts and applied that research and theory to both personal reflection and immediate integration into teaching and learning. Throughout the course, participants were asked to experiment with key concepts and practices, such as structured uncertainty, metaphorical thinking, divergent idea production, and active reflection. Teachers were prompted to consider their journey through the Foundation Course as if it were a river journey, illustrating how metaphors can be a gateway into creative thinking and meaning-making. For instance, early in the course, teachers were presented with the scenario that they had just begun their river journey only to realize they had forgotten sunscreen. They would need to come up with solutions for how to protect themselves from the hot sun overhead. Participants were encouraged to think of divergent and unusual ideas. Throughout the course, teachers were asked to reflect on their process and the emotions they experienced using different modalities (e.g., writing, sculptural, and gestural). Teachers were able to download protocols to integrate and expand the creative and reflective prompts and routines into their classrooms and content areas.

#### Virtual Summer Institute

The 2 day virtual Summer Institute was hosted on Zoom videoconference software and through the learning management system where the foundation course was accessed. The experience provided synchronous presentations from facilitators and pre-recorded presentations that participants watched and reflected on through a discussion forum. All participants had access to those discussion forums and could read and respond to their peers’ posts. The synchronous and asynchronous activities were hands-on and integrated different creative routines, active reflections, and arts integration strategies that the foundation course introduced. With foundational understanding set through the online course, the Summer Institute established an open attitude for risk-taking and shared creative experiences. By practicing simple creative and artistic routines with facilitated support, teachers were encouraged to bring these routines back to their classrooms. Teachers’ final forum posts at the Summer Institute included their adaptations and ideas to engage students’ creatively in distance learning early in the 2020–2021 school year. Those posts were analyzed to respond to research question 4. Teachers started the Foundation course before the Summer Institute and finished after the institute.

### Measures

#### Affect and Well-Being

This study used or adapted measures validated in past research to study perceptions, beliefs, and experiences related to psychological, affective, and creative phenomena. We studied the psychological well-being of teachers using the Positive and Negative Affect Scale (PANAS) (Watson et al., 1988). For the PANAS, we adapted the prompt to fit the teaching context: “Indicate to what extent you have felt this way generally in your work as a teacher this past year (e.g., interested, distressed, excited, etc.).” Similarly, we adapted the prompt for the Secondary Traumatic Stress Scale (STSS) from Bride et al. (2004) to focus on the teaching context during the past term of school, using an average score that included the subscales of *intrusion, avoidance*, and *arousal*; a sample STSS item was “It seemed as if I was reliving the trauma(s) experienced by my students.” The PANAS and STSS were measured on a 1–5 modified frequency scale where 1 = never and 5 = very often (see Supplementary Appendix for all items). We assessed the dispositional state of joy in teaching that teachers experienced during the 2020 spring term of distance learning using the dispositional joy scale with slight adaptations to be directed at teachers (Watkins et al., 2017). For instance, one sample item was “Many things about being a teacher bring me delight.” We modified the Academic Buoyancy Scale, developed by Martin and Marsh (2008) to focus on the experience of setbacks typical to teachers (e.g., *I don’t let teaching stress get on top of me*). Those scales were on a 1–6 Likert scale for agreement with 1 = strongly disagree and 6 = strongly agree. Items for all scales are included in Supplementary Material.

#### Creative Resources

We used the Creative Self-Efficacy in Teaching scale (e.g., *I feel that I am good at coming up with novel ideas for teaching*), used in past research with similar samples (e.g., Anderson et al., under review). Additionally, tolerance for ambiguity in teaching is an important affective and attitudinal factor underlying creative potential, potentially stimulating openness to creative opportunities as they arise naturally (Lubart et al., 2013). Tolerance for ambiguity was assessed with original items modified to the teacher context from Need for Closure scale (Kruglanski et al., 2013) and with two new items. That scale included five items targeting teachers’ *need for closure facing ambiguity in teaching*. A slightly modified original item was “When teaching, I don’t like situations that are uncertain.” A new item added to enhance reliability was “I feel discomfort when a student asks me questions I don’t know the answer to.” Additionally, we used the Environmental Support and Encouragement for Creativity subscale (Rubenstein et al., 2013) to understand the role of environmental support and encouragement for creativity around the teacher (e.g., “My administration encourages me to foster innovative thinking in my students”). A creative fixed mindset scale (e.g., *To be honest, I can’t really change how creative I am*) and a creative growth mindset scale (e.g., *I can always change or increase my creativity in instruction*), used in past research with similar samples, were used (Anderson et al., under review; Hass et al., 2016). All scales, items, and Cronbach’s alpha for internal consistency are included in Table 2 and Supplementary Appendix.

**TABLE 2:** Descriptive results for teachers’ psychological well-being, affect, and creative resources during the 2020 spring term of distance learning (*N* = 35).

Teacher factor	α	Mean (*SD*)	Min	Max
Creative self-efficacy in teaching	0.89	4.27 (0.79)	2.67	6.00
Environmental support for creativity	0.80	3.82 (0.94)	1.40	6.00
Growth creative mindset	0.92	5.24 (0.67)	3.50	6.00
Need for closure facing ambiguity in teaching	0.80	2.74 (0.87)	1.20	5.40
Fixed creative mindset	0.89	1.67 (0.67)	1.00	3.00
Creative anxiety	0.87	2.12 (0.83)	1.00	4.00
Experience of positive affect	0.91	4.10 (0.54)	2.75	5.00
Dispositional joy in teaching	0.94	4.56 (0.75)	2.89	6.00
Buoyancy in teaching	0.93	3.82 (0.98)	1.00	6.00
Experience of negative affect	0.84	2.51 (0.77)	1.00	4.50
Secondary traumatic stress–avoidance, intrusion, arousal	0.94	2.55 (0.88)	1.00	5.00

*Creative self-efficacy, environmental support, growth and fixed creative mindsets, need for closure, dispositional joy, and buoyancy subscales are on a 1–6 response scale from “disagree” to “agree.” secondary traumatic stress are on a 1–5 frequency scale from “never” to “very often.” Experience of positive and negative affect and creative anxiety subscales are on a 1–5 emphasis scale from “very slightly” to “extremely.”*

#### Quantitative Analytic Approach

Using SPSS version 26 software, we analyzed and reported descriptive data, internal consistency, bivariate correlations between all factors, and regression diagnostics and results. Once the strongest correlations were detected between creativity and well-being factors, we prepared to run multiple regression models to identify the creativity factors (IVs) that contributed most to aspects of well-being (DVs). Prior to running multiple regression models, we tested assumptions of multicollinearity and homoschedasticity and report those findings in the section “Results.”

### Teachers’ Descriptions of Stressors and Creative Ideas for the Classroom

To respond to research questions 3 and 4, data were analyzed from teacher responses to two open-ended items: (a) *How has the COVID-19 pandemic affected your capacity to provide creative opportunities for your students?* and (b) *How has the COVID-19 pandemic and distance learning caused you more stress than usual in your work as a teacher? Have you done anything, specifically, to reduce that stress?* The 2020–2021 school year began for all participating teachers in a fully distance learning environment. Teachers responded to the prompt, *What activities or creative opportunities can you imagine offering your students during the first 2 weeks of school (e.g., helping them feel seen but not exposed, celebrating a competency they carry, or setting conditions for creative engagement).*

#### Qualitative Analytic Approach

Data analysis focused on describing units of meaning that recurred within and across each teacher’s written reflections and instructional plans (Lincoln and Guba, 1985; Merriam, 2009). One author analyzed each of the data associated with one of the three prompts described above, which included the brief responses from all participating teachers. To analyze the data and write descriptive results, each author (a) grouped teachers’ responses within thematic codes that emerged from the data inductively during the first screening of the data; (b) read through the data a second time to ensure saturation of themes; and (c) compiled teachers’ reflections and ideas within each theme identifying a representative quotation to include in the text.

## Results

Following the explanatory sequential mixed methods design (Creswell and Plano Clark, 2018), we first present the quantitative results and then describe the qualitative results and how they explain and extend the quantitative results.

### Quantitative Results

The initial results from descriptive statistics in Table 2 and bivariate correlations in Table 3 illustrate connections between creative beliefs and affect and psychological well-being in the experience of teaching during the COVID-19 pandemic. For adaptive creative beliefs and support, based on a 1–6 Likert scale, teachers, on average, demonstrated a high level of growth creative mindset (*M* = 5.24; *SD* = 0.67), a moderate level of creative self-efficacy in teaching (*M* = 4.27; *SD* = 0.79), and a moderate level of environmental support for creativity in school (*M* = 3.82; *SD* = 0.98), which was lowest among creative resource factors but still above the scale midpoint. For maladaptive creative affect and beliefs, on average, teachers showed a low need for closure facing ambiguity in teaching (*M* = 2.74; *SD* = 0.87 on a 1–6 Likert scale), low creative anxiety (*M* = 2.12; *SD* = 0.83 on a 1–5 frequency scale), and low fixed creative mindset (*M* = 1.67; *SD* = 0.67 on a 1–6 Likert scale). Regarding general well-being in teaching, teachers reported, on average, moderate frequency of positive affect (*M* = 4.10; *SD* = 0.54 where 4 = quite a bit), moderate-to-high levels of joy in teaching (*M* = 4.56; *SD* = 0.75 on a 1–6 Likert scale), and lower levels of buoyancy in teaching (*M* = 3.82; *SD* = 0.98 on a 1–6 Likert scale). Teachers reported moderate frequency of both negative affect in teaching (*M* = 2.51; *SD* = 0.77) and the avoidance, intrusion, and arousal of secondary traumatic stress (*M* = 2.55; *SD* = 0.88) on a 1–5 frequency scale.

**TABLE 3:** Bivariate correlations between factors of psychological well-being, affect, and creative resources.

Variables	1	2	3	4	5	6	7	8	9	10	11
1. Creative self-efficacy in teaching	–										
2. Environmental support for creativity in school	0.11	–									
3. Growth creative mindset about self	0.23*	0.07	–								
4. Need for closure with ambiguity in teaching	−0.42*	0.04	–0.13	–							
5. Fixed creative mindset about self	–0.07	0.09	−0.56*	0.14	–						
6. Creative anxiety	−0.35*	0.06	−0.28*	0.50*	0.29*	–					
7. Experience of positive affect	0.18	0.31*	0.38*	0.05	–0.15	–0.13	–				
8. Dispositional joy in teaching	0.24*	0.33*	0.21	–0.11	–0.12	–0.15	0.51*	–			
9. Buoyancy in teaching	0.35*	0.22	–0.12	–0.12	0.12	–0.13	0.19	0.51*	–		
10. Experience of negative affect	–0.16	−0.28*	0.13	0.16	0.05	0.25*	–0.06	−0.30*	−0.46*	–	
11. Secondary traumatic stress–Arousal, intrusion, and avoidance	–0.14	–0.18	–0.13	0.32*	0.13	0.58*	−0.31*	−0.31*	−0.33*	0.43*	–

*p < 0.10.

#### Relationships Between Creativity and Positive and Negative Affect

In terms of links between creative beliefs, affect, and environmental support and general psychological well-being in teaching, relationships fit within the theoretical perspective that creative resources should buffer against the stress of uncertainty and instability. As Table 3 illustrates, meaningful links between creativity factors and well-being were generally distinct with few overlapping relationships. Three well-being outcomes demonstrated overlapping relationships with two factors of creativity. Once multiple regression was conducted, all but one well-being factor was predicted by a single unique creativity factor. Effect sizes are reported for both correlations and multiple regression models according to Cohen (1992) suggested index for *r* (small effect = 0.10, medium effect = 0.30, and large effect = 0.50) and *R*^2^.

#### Adaptive Creative Beliefs and Environmental Support

Creative self-efficacy in teaching demonstrated medium effect size positive correlations with buoyancy in teaching (*r* = 0.35, *p* < 0.05) and dispositional joy in teaching (*r* = 0.24, *p* < 0.10). Environmental support for creativity in school demonstrated a medium effect size positive correlation with dispositional joy in teaching (*r* = 0.33, *p* < 0.05) and general positive affect in teaching (*r* = 0.31, *p* < 0.05), such as feeling interest, enthusiasm, pride, inspiration, and determination, and a medium effect size negative correlation with general experience of negative affect in teaching (*r* = −0.28, *p* < 0.05), such as feeling irritable, guilty, upset, or nervous. Growth creative mindset only demonstrated a medium effect size positive correlation with general positive affect in teaching (*r* = 0.38, *p* < 0.05), alongside environmental support. Only self-efficacy for one’s creative teaching ability related to the buoyancy to bounce back from difficult challenges and setbacks in teaching.

Standardized beta coefficients are reported in text, and both unstandardized and standardized coefficients are reported in Table 4. For the first model, we regressed the experience of general positive affect in teaching onto two adaptive creativity factors—environmental support for creativity and growth creative mindset. According to diagnostic statistics, no issues of heteroscedasticity, multicollinearity, or non-normality were present. Both environmental support for creativity (*b* = 0.289, *t* = 2.41, *p* < 0.05) and growth creative mindset (*b* = 0.359, *t* = 2.99, *p* < 0.05) remained significant predictors of general positive affect. As a model, the explained variance in general positive affect was *R*^2^ = 0.23, nearly a large effect size. For the second model, we regressed dispositional joy onto environmental support and self-efficacy for creative teaching. According to diagnostic statistics, no issues of heteroscedasticity, multicollinearity, or non-normality were present. Environmental support remained statistically significant (*b* = 0.30, *t* = 2.40, *p* < 0.05), and creative self-efficacy did not (*b* = 0.21, *t* = 1.65, *p* > 0.10); however, that result may be due largely to sample size restrictions. As a model, the explained variance was *R*^2^ = 0.23, nearly a large effect size. Self-efficacy for creative teaching played a unique role, only, in teacher buoyancy; environmental support played unique roles in teachers’ positive affect and joy, and growth creative mindset played a role in teachers’ positive affect. Except for environmental support, no other adaptive creativity factor played a role in secondary traumatic stress and or experiences of negative affect in teaching (reported below).

**TABLE 4:** Multiple regression model results and collinearity statistics.

Predictors	*B* (*SE*)	*b*	Tolerance	VIF
Model 1: Positive affect				
Constant	1.19* (0.74)	–	–	–
Environmental support	0.18* (0.08)	0.29	1.00	1.00
Growth creative mindset	0.13* (0.13)	0.36	1.00	1.00
Model 2: Dispositional joy				
Constant	2.39* (0.67)	–	–	–
Environmental support	0.26* (0.11)	0.30	0.99	1.01
Creative self-efficacy in teaching	0.21 (0.13)	0.21	0.99	1.01
Model 3: secondary traumatic stress				
Constant	1.29* (0.34)	–	–	–
Creative anxiety	0.58* (0.13)	0.55	0.75	1.34
Need for closure facing ambiguity	0.05 (0.13)	0.05	0.75	1.34

Tolerance and VIF are diagnostics of collinearity, where a VIF substantially greater than 1 and Tolerance below 0.2 indicates potential bias (Bowerman and O’Connell, 1990; Menard, 1995). *Denotes *p* < 0.05.

#### Maladaptive Creative Affect and Beliefs

**FIGURE 3:**
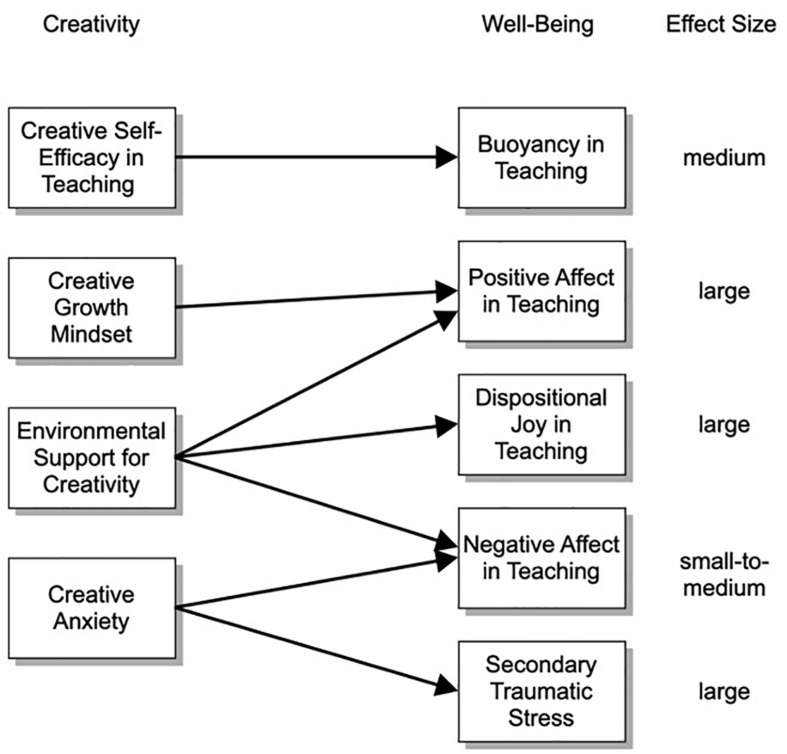
The combined results of multiple regressions for each well-being outcome conducted in this study, showing relationships between creativity and well-being factors and the associated effect size for each model.

Fixed creative mindset did not demonstrate a statistically significant relationship with any factors of well-being. Teachers’ need for closure when facing ambiguity in teaching demonstrated a medium effect size positive correlation with secondary traumatic stress in teaching (*r* = 0.32, *p* < 0.05). Creative anxiety demonstrated a large effect size positive correlation with secondary traumatic stress in teaching (*r* = 0.58, *p* < 0.05) and medium effect size positive correlation with general negative affect in teaching (*r* = 0.25, *p* < 0.05). For the third regression model, we regressed secondary traumatic stress onto the two interrelated maladaptive factors, creative anxiety and need for closure when facing ambiguity. According to diagnostic statistics, no issues of heteroscedasticity, multicollinearity, or non-normality were present. Creative anxiety (*b* = 0.55, *t* = 4.32, *p* < 0.05) remained a statistically significant predictor of general stress, but need for closure did not (*b* = 0.05, *t* = 0.35, *p* > 0.05). As a model, the explained variance in general negative affect was *R*^2^ = 0.34, a large effect size. With a *r* = 0.50 correlation between creative anxiety and need for closure, that result clearly shows that the relationship between need for closure and negative affect in teaching was explained by the overlap it shares with creative anxiety. As such, general creative anxiety appears to play a substantial role in the experience of secondary traumatic stress in teaching. When regressing teachers’ negative affect onto environmental support for creativity in school and creative anxiety, environmental support (*β* = −0.23, *t* = 2.31, *p* < 0.05) and creative anxiety (*β* = 0.24, *t* = 2.09, *p* < 0.05) remained statistically significant predictors, and the model explained variance in general negative affect of *R*^2^ = 0.15, a small-to-medium effect size. The maladaptive factor of creative anxiety demonstrated a negative relationship with creative self-efficacy in teaching (*r* = −0.35) and growth creative mindset (*r* = −0.28), but it did not play a role in general positive affect, buoyancy, or joy experienced in teaching. As expected, need for closure when facing ambiguity in teaching demonstrated a negative relationship with self-efficacy for creative teaching, but it did not play a role in the experiences of positive affect, buoyancy, and joy experienced in teaching. The model of results is illustrated in Figure 3.

### Qualitative Results

#### Diminished Opportunities for Creativity

According to teachers’ open-ended responses on the survey, the COVID-19 pandemic negatively affected their capacity to provide creative opportunities for their students, in most cases. Thirteen out of 57 teachers (23%) held an adaptive and positive perspective about the effects of the COVID-19 shutdown in terms of their capacity to provide creative opportunities to their students during the spring term of the 2019–2020 school year. Below, the negative and positive effects of the pandemic are discussed within the following themes: (a) student disengagement; (b) disconnection through the virtual format; (c) issues of equity, trauma, and stress; and (d) being forced to be adaptive and try new techniques.

#### Student Disengagement

Several teachers bemoaned the frustration of offering students new and creative ways to engage with the learning material with little to no follow-through and interest. For instance, one teacher shared,

“I’ve offered students the opportunity to handwrite, draw/sketch, color, cut, glue, and/or act out various assignments and then take a picture or video their work; however, most students have opted to just type out their ideas. I’m not sure if it was due to a lack of my instruction/examples, lack of materials at home, access to creativity due to stress, or simply disinterest (White female high school teacher with 24 years teaching experience.)”

Several teachers felt like students probably struggled with using the online platforms, establishing consistent online connectivity, and finding interest and accountability in academic work. Teachers believed students lacked the kind of hands-on work crucial for facilitating deeper engagement in classes.

#### Issues of Equity, Trauma, and Stress

Teachers perceived a “dramatic drop in equity,” as one teacher shared, and felt unable to ensure students had the proper materials, supplies, and tools to be successful. Several teachers felt like the school shutdown likely affected students with special needs the hardest. One teacher shared the quality of student work and evidence of skill development was far weaker than prior years in his woodcarving class because he was unable to provide the same level of oversight and support. Teachers were unable to engage students in the socially vital process of collaborative learning, which teachers felt limited creativity for some.

Several teachers felt that distance learning needed to be a whole different approach. Most students had been traumatized by the pandemic, to some degree, and the stress teachers experienced themselves in their homes and the secondary stress they absorbed from their students kept them from feeling safe enough to be creative. As one teacher shared, “I’m not sure that I did much that was creative. We were all just trying to survive” (Hispanic female middle school teacher with 20 years teaching experience). Several teachers reported feeling stifled, stressed, and stunted in their creativity. However, one teacher shared that even though distance learning was stifling at first, upon reflection, it may have supported their growth.

“During distance learning my capacity to provide creative opportunities for my students was non-existent. Now that I have time to reflect and look back on the experience I feel like my creative options going into this school year has grown. Change is uncomfortable, and distance learning was very very uncomfortable, but I feel as though it has changed my capacity to provide creative opportunities for the better. We will see (Hispanic female elementary school teacher with 2 years teaching experience.)”

#### Disconnection Through the Virtual Format

The virtual format of distance learning meant some teachers felt they could not read students’ facial expressions, pick up on the nuances of their body language, develop relationships with new students, or support students emotionally. Some teachers felt the screen-based connection limited classroom discourse, how students could show their learning, and teacher guidance. For art teachers, the distance learning format posed especially difficult conditions to support students’ creative expression and skill development.

In addition to distance learning challenges, several teachers felt like the online system they were using focused on “getting down to business to get all the standards met.” As one teacher shared, “The double whammy of COVID-19 and my district going to several scripted curriculum with a rigid master schedule has completely bled dry my creative teaching side” (white female elementary school teacher with 16 years teaching experience). One teacher expressed disappointment to let go of plans to act out *The Lion, Witch, and the Wardrobe* in Spanish; however, as described further below, some teachers found distance learning challenges enhanced creativity in some ways.

#### Feeling Forced to Be Adaptive and Try New Techniques

In 23% of cases, teachers identified some benefits to being forced to adapt to the distance learning format. The circumstances appeared to demand their creative resources in unexpected ways. “COVID-19 has forced me to become more creative,” one teacher wrote. For another, the pandemic gave permission and freedom to break from the highly scripted curriculum, which actually allowed for more creative learning opportunities for students. Another teacher noted that many students had fewer creative opportunities because of factors outside of their control, but others engaged with online tools and communication in a way that enhanced their creativity. As one teacher put it,

“Distance learning required students to use different things, learn in a different way, so in a way excited their creativity in that way. I tried to find different types of assignments and ways of doing assignments. Everything was new for all of us (White female middle and high school teacher with 7 years teaching experience.)”

Another teacher suggested that COVID-19 was actually not the most limiting factor for creative learning opportunities: “My [school] admin is more problematic.”

One teacher embraced the challenge and shared in detail the kinds of creative maneuvering she attempted for her elementary school students when schools shut down.

“I gathered numerous items to give to my students in order to keep them engaged and learning in daily zoom meetings. These included scarves and balls for juggling, bandanas and hair ties to teach mask-making, pumpkin seeds and cups for a growing contest, permanent markers and a 16 × 16 piece of oak with drawn grids to color checkerboards, supplies for making tetrominoes and tesselations, books for reading together, and whiteboards and markers for answering questions and playing Pictionary. Daily zooms also included scavenger hunts, online games, show and tell, PE, and new websites (White female elementary school teacher with 23 years teaching experience.)”

For a quarter of the teachers, the pandemic may have actually enhanced their creative resources. The opportunistic and adaptive attitude among this segment of participants was summed up well in the following teacher reflection: “[COVID-19] has upended the whole works—while stressful, it is also an opportunity to make lasting and dramatic changes” (white male middle and high school teacher with 22 years teaching experience).

#### Stresses and Stress Relief During the COVID-19 Pandemic for Teachers

According to teachers’ open-ended reflections, the COVID-19 pandemic increased their stress at work. Out of the 57 respondents, very few reported not having any increased stress in their work as a teacher due to the pandemic. More typically, teachers responded that the pandemic and accompanying shift to distance learning created greater stress for teachers for a variety of reasons. The most common reason was the lack of direct connection to their students in the physical classroom environment, whether to increase support and accountability for completing assignments or just having the social interaction and engagement. As one teacher shared, “not having my students in front of me where I can better read their feelings/engagement has been limiting for me. While teaching in a classroom setting, I feed off of/react/riff/improvise based on my students’ energy, interest/lack of interest, and questions.” Another teacher reported, “I have felt that I abandoned my students because I was not able to help them in the ways I wanted to. This caused me a lot of stress” (white female middle school teacher with 23 years teaching experience).

The second most common stressor reported by teachers was the general lack of certainty that accompanied the COVID-19 pandemic and distance learning. For some, the uncertainty about what school would look like when they returned to the classroom for the 2020–2021 school year was a source of anxiety, while others noted a more generalized sense of anxiety about the pandemic’s effect on their lives and others. As one teacher shared, “…stress and anxiety wasn’t something I typically struggled with pre-COVID. The unknowns are causing lots of this on a fairly regular basis. There is no summer break for teachers. We are being asked to be prepared for all of the possible outcomes for reentry in the fall” (white female high school teacher with 8 years teaching experience). The feeling of uncertainty intensified for many teachers. One teacher shared, “the most stress has been caused by not knowing what is going to happen in school, the state, the nation, and world from day to day. We are in the middle of summer and still do not know what we will be doing in a month and a half as far as school goes” (white male elementary school teacher with 8 years teaching experience).

Several teachers reported that the increased workload and expectations in moving work to the remote learning environment and needing to create new online learning materials was their biggest stressor, as well as having to learn new technology and delivery modes. Many teachers noted that they worked far out of their normal workday in trying to maintain communication with colleagues, students, and parents. One teacher shared, “the distance learning aspect has caused much more stress in my life. I’ve worked very hard to do the best for my students. My hours of availability started at 6 am and went past 9 pm, at times. When a student had a question, I made myself available” (elementary school white male teacher with 8 years teaching experience). Only four of the respondents reported identifying as Hispanic/Latinx, so it was noteworthy that one respondent felt additional stress due to their ethnicity, which may suggest similar consequences for other teachers marginalized by their identity during the pandemic.

“Currently, I am stressed about the politics that surround every aspect of this pandemic. I am Hispanic and I was born and raised in this small town. I have seen it grow and little by little become a place that is more welcoming to minorities then it was before. Now, looking at our town’s Facebook pages, I am left wondering if this was just a facade or if we only have a few very vocal bad apples that want to simply stir things up. What hurts the most isn’t the initial posts but the lack of people who point out these biases and misconceptions (Elementary school white female teacher with 2 years teaching experience.)”

Teachers reported a variety of approaches to dealing with this new level of stress. Of the respondents who reported taking steps to reduce their stress, most included a mention of increasing or maintaining their physical activity in some way, such as walking, running, or other types of exercise routines and movement breaks. Many also reported using practices such as meditation, yoga, journaling, reading, and other calming and mindfulness routines. Several respondents described picking up a new creative hobby or returning to an older one, such as drawing or writing. A few teachers noted that they reduced their stress by being more protective of their work hours and/or creating a home office space dedicated to work. Additionally, several teachers said they are trying to use their time to become more creative in their teaching and looked forward to bringing new skills and activities back to the classroom. Teachers also took time to talk to family, friends, and other educators to deal with stress.

#### Teacher Adaptations and Innovations for Distance Learning

Teachers were led through a synchronous virtual Summer Institute late in the summer of 2020, after completing most of the Foundation Course. In the Summer Institute, they used a learning management system to engage in self-directed content and videoconferencing software to listen to presenters and interact with others, synchronously. As part of this process, participants created activities or adapted creative teaching and learning strategies for their own grade level and content area. Some foundation course participants were not available for the Summer Institute. As such, 44 teachers shared 52 discrete activity plans they intended to implement during the 2020–2021 school year. Of those activities, 41 were directly related to or inspired by the material from the course and Summer Institute and all were designed to support students’ creativity and growth. The remaining 11 activities emerged explicitly from teachers own inventiveness, potentially as a result of their creative development through the trainings.

#### Using Creative Routines to Develop Community and Sense of Belonging

Of the total activities, 37 or 71%, of the teacher-developed activities were designed to build classroom community and instill a sense of belonging through creative and metaphorical thinking, including the following: using metaphors to help students feel like a team who are collaboratively tackling challenges together, asking students to introduce themselves in creative ways and respond to one another, and offering opportunities for students to individually contribute to a shared creation through distance learning. Inspired by the course-level *river journey* metaphor designed into the foundation course, six teachers planned to use an outdoor exploration or adventure to create a story for their curriculum and classroom community.

In one example, students and their teacher would form an expedition team that prepares for and faces challenges, growing their skillset along the way. “Before we can go on any hike, we must get to know our fellow hikers,” a teacher wrote. To this end, in her classroom “the first 2 weeks will be building relationships with each other.” Another teacher planned to start the school year by “cautioning students that this is new territory, an unexplored mountain range, and it *will* be difficult, but we have each other and we will be *ok*.” Teachers described the journey through this uncertain school year as a collaborative process, working through challenges and growing as a team. Teachers were employing these macro-level metaphors for the upcoming school year as a way for their classes to build story together, generate additional metaphors within this large one, and model to provide permission for students’ creative thinking.

In other proposed adaptations, students and their teacher would work together to create a shared work of art. Many teachers decided to specifically and intentionally address the challenges of building community in a virtual environment. One teacher sought to “[build] back our community in a tangible way, so that people can see their distanced selves brought together into a coherent whole.” This fall, her students will create living “exquisite corpses” in small Zoom Breakout Rooms by assembling their bodies in creative ways using the Zoom grid layout. Her class will also create a “Zoom quilt” when students individually strike poses on video conferencing to create the composite piece. Similarly, another teacher planned to ask students to use gesture to demonstrate an activity they enjoyed during the weekend and taking a screenshot of the “quilt.” Next, students would try to guess what others communicate through their poses. These creative routines served as starting places for more elaborate creative opportunities.

Asking students to share experiences, skills, preferences, and dreams was common to many of the developed activities. Teachers hoped to learn more about their students and to encourage them to learn more about each other. One teacher combined two Foundation Course techniques to create a lesson plan. Early in the fall, her students would each create an avatar that communicates some aspects of who they are. Classmates would react to others’ avatars using the “I notice…, I wonder…, I appreciate…” protocol for reflective appreciation and critical thinking. Another teacher would assign small groups to use the Whiteboard feature in Zoom Breakout Rooms to draw their individual hopes for the year on the same page. Next, they would present their groups’ hopes to the rest of the class.

#### Using Creative Routines to Build Autonomy

Student autonomy can be especially difficult to develop in an online learning environment, and participating teachers were determined to find ways for students to express themselves, reflect on what makes them unique, and grow their independence. In total, 61% of developed activities were designed specifically to cultivate student autonomy. In addition to sharing their attributes, experiences, and favorite things with others in the class, teachers planned to invite students to reflect privately, through journaling, drawing, generating gesture, or using objects metaphorically to tell a story or share about a feeling or experience.

Four teachers were inspired by a song-based video describing how everyone has different virtues. They shared they would ask students to identify and explore their own virtues. One explained, “I really think it’s important for students to develop a sense of themselves.” Another would assign students to identify “anchors” in their lives—people who provide needed support—and consider what virtues they have. After identifying that person’s strengths, students would create a metaphor for that person, write about how that person’s virtues shine through actions, and consider the potential of their own virtues in their lives and the world around them.

Drawing a *selfie*, or a quick doodle to represent a feeling or attribute among many other ideas, is another common tool teachers adapted to help students explore their strengths and attributes. One teacher planned to instruct students to draw selfies in response to specific prompts, such as, “What is your ideal self?” and “What is one goal you can accomplish within the next week?” Teachers planned to prompt students to use selfies to provide an update on the goal and a reflection on the process of setting and working toward the goal. Similarly, two teachers who planned activities about virtues would ask students to reflect on new virtues they develop throughout the school year and reflect on their growth.

#### Using Creative Routines to Support Well-Being

According to teachers, distance learning and the conditions of a global pandemic that precipitated school closure caused distress for students. The difficulty for teachers to connect and care for students only exacerbated the issue. One teacher lamented, “Checking in with students is so much harder no[w] that we are not face-to-face.” In total, 25% of developed activities specifically aimed at supporting students’ mental health and emotional well-being. One teacher explained, “Learning where the kids are at and how they are feeling through art will help them process the difficult circumstances many of my students and their families face.” She planned to use self-portraits and “Who Am I?” and “How am I Feeling?” journaling activities in her classroom this fall. Another teacher created the metaphorical *Drop Off Your Luggage* activity to enable students to regularly reflect on their heaviest thoughts, feelings, and experiences. Students would write one or two ideas down on a piece of paper, put that paper on the floor, and let the teacher or class know what they need “to keep that thought dropped off” for the remainder of the class. Students would not be asked to share specifics about their “luggage,” but they would be asked to let the teacher know if they need a “check-in afterward, a distraction, space, [or] compassion.”

Similarly, another teacher planned to implement a daily activity called the *Mental Health Check-in*. “Students complete a form that has the metaphor cards on it and they are asked to relate to one of the cards and describe why.” *Metaphor Cards*, a technique presented in the Foundation Course, help students generate metaphor about any abstract idea or feeling by selecting an object or scene from a variety of cards that best represents what they are thinking about. The teacher explained, “I am prioritizing the check-ins because it is such an important part of my job. I teach middle school students and they are faced with so many pressures and changes every day and sometimes they are not sure how to express themselves.” This difficulty is exacerbated by current events and distant learning, which only reinforces the need for mental and emotional awareness and care.

## Discussion

The results of this mixed methods study shed light on two aspects of the teacher experience in the COVID-19 pandemic. First, quantitative results specify the relationships between teachers’ creative beliefs and affect and their well-being in teaching during the early phase of the COVID-19 shutdown. Second, qualitative results indicate the effects of the shutdown on creative opportunities for their students, causes of teacher stress, and the creative adaptations teachers generated to enhance distance learning based on 30 h of training.

### Integrating Quantitative and Qualitative Results

Quantitative results highlight specific relationships important to future research and development focused on teacher creativity and well-being during times of crisis. Because these analyses are correlational and cross-sectional, directionality of any potential influence remains inconclusive. It is plausible and likely, in some cases, that factors of well-being have a reciprocal effect on creative beliefs, affect, and attitudes. (a) Creative self-efficacy in teaching may support teacher buoyancy in the face of setbacks. (b) Creative growth mindset may support teachers’ general positive affect in teaching. (c) Lowered creative anxiety may support the reduction of secondary traumatic stress and general negative affect in teaching. (d) Environmental support and encouragement for creativity in schools may be foundational for teacher well-being by enhancing teachers’ dispositional joy, general positive affect, and reducing general negative affect. Importantly, perceived support was unrelated to key creative beliefs and affect.

Qualitative results helped to clarify and extend these findings in important ways. Given that 23% of teachers actually felt the pandemic enhanced their creativity in teaching based on their open-ended responses and the conditions under which teachers selected into the program, it may not be surprising that teachers reported high levels of adaptive creativity factors and low levels of maladaptive factors in the quantitative results. For instance, this cohort of teachers held higher creative growth mindset and lower creative fixed mindset than another similar sample of teachers in other research (Anderson et al., under review). Generally, teachers found that COVID-19 and distance learning had negative effects on their capacity to build supportive relationships with students, on students’ equitable access to creative opportunities, and on the overall well-being and academic engagement of students. The majority of teachers experienced greater stress and a stifling and stunting of their creative potential in teaching.

Qualitative responses aligned with the quantitatively derived relationships between creative self-efficacy and buoyancy in teaching, between creative growth mindset and positive affect in teaching, and between creative anxiety and secondary traumatic stress. The confidence and willingness to take risks in the face of such a massive creative challenge as distance learning related to teachers’ well-being. If teachers felt anxious to have to think of new ways to reach and engage students, they also experienced greater stress, including how secondary traumatic stress aroused feelings of guilt and hopelessness, as if they had abandoned their students. In this way, approaching the challenge with a creative growth mindset, lower anxiety, and greater self-efficacy may be protective of teacher well-being.

As the quantitative results suggested, supporting and encouraging creative development of teachers during times of crisis and uncertainty, such as distance learning due to the COVID-19 pandemic, could be a key to unlock adaptability and resilience to meet this extraordinary demand. Survey items for environmental support targeted the school placing high value and priority on creative development of students (Rubenstein et al., 2013). When teachers felt this support, their overall well-being and joy was also higher. Teachers did not suggest much collaboration among colleagues in the open-ended responses about the experience in the early stages of the school shutdown. However, in several cases, the creative adaptations generated at the conclusion of their summer training included collaborations across multiple teachers to generate more consistent creative opportunities for students. The focus of their adaptations also honed in on the social and emotional wellness of their students or focused on building community and shared story through playful metaphors about their experience together.

### Training Teachers to Be Creative (and Less Stressed) in Crises

All participants who were available to engage in the virtual Summer Institute received the support, modeling, and guidance to ideate creative adaptations for distance learning that would engage their students’ creativity and support social-emotional wellness. Described earlier, the Foundation Course focused on teachers’ understanding and beliefs about creativity with the aim of establishing adaptive beliefs and attitudes toward creative challenges by scaffolding creative thinking and behaviors through basic routine exercises. Extensive research from international contexts highlights the powerful role teachers’ beliefs about creativity play in actual creative teaching and learning in the classroom (Bereczki and Kárpáti, 2018). A previous study looking at teacher change and classroom implementation (pre-pandemic) suggests this approach to training may be highly effective (Anderson et al., under review). Based on teacher adaptations in this current study, it appears important to make any type of training, whether virtual or in-person, a mix between theoretical and experiential, offering adaptive beliefs and perspectives alongside routines and exercises that can be applied immediately. Given the overarching role of environmental support for creativity, it may be important for administrators to engage in this kind of training, as well, to increase awareness, understanding, and encouragement.

Conceptually related to creative anxiety, higher tolerance for ambiguity may help reduce the effects of secondary traumatic stress, but results indicate that any unique contribution of tolerance for ambiguity on lower stress was explained by general creative anxiety. Creative anxiety arises when facing undefined and unstructured challenges, which conceptually relates to how teachers face and manage the potential stress of caring for students experiencing trauma. Secondary traumatic stress includes the emotional arousal, intrusion of stress into daily life, and avoidant behaviors related to that stress (Bride et al., 2004). It is plausible that being able to deal with the anxiety of having to respond creatively in moments of uncertainty could produce adaptive responses that would be relevant to managing the intrusion, arousal, and avoidance of secondary traumatic stress. For instance, teachers who work with students facing issues of homelessness may find creative ways to support students and remain mindful of what they can and cannot control. Creative action can be considered agentic and metacognitive (Karwowski and Beghetto, 2018; Anderson and Haney, 2020), so it makes sense that other creative resources, such as associative and divergent thinking, beyond those included in this study, could facilitate management of this kind of stress for teachers. These results are only cross-sectional and correlational and need more rigorous longitudinal analyses to understand unique contributions of each factor, stability across time, effects of interventions, and mechanisms of change.

### Future Directions

Reviewing the quantitative results from this study illustrates a theoretical model about creative beliefs, affect, and attitudes and general teacher well-being that should be studied further. For instance, past research suggests that buoyancy in the face of typical setbacks for students mediates the role of predictive factors, such as self-efficacy, and the resilience to face more chronic forms of adversity (Martin and Marsh, 2009). In this current study, buoyancy in teaching demonstrated a substantial positive relationship with teachers’ joy and a negative relationship with negative affect and secondary traumatic stress. Incorporating those results with prior research presents a model where creative self-efficacy in teaching supports teacher buoyancy which, in turn, reduces negative affect and stress and boosts joy—especially during times of great uncertainty. Longitudinal research will be needed to test this model, empirically.

Though correlational, results suggest that lower creative anxiety may help buttress teachers from the negative emotional experiences and secondary traumatic stress faced in teaching during crises like the COVID-19 pandemic. Research about creative anxiety is still very new (Daker et al., 2019) and suggests that it may be more pronounced across domains for women. To date, no interventions have been established to address this anxiety. It is plausible that helping teachers gain control over this anxiety could have a positive effect on how they manage crises of different magnitude and the complex stress experienced by caring for students managing trauma. It will be important to learn how training and interventions for teachers can assuage this form of anxiety, effectively.

If teachers perceive their school to value and prioritize students’ creative development, they also appear to experience more joy in teaching and other types of positive affect, such as interest, enthusiasm, pride, inspiration, and determination. Moreover, school climate that encourages students’ creative thinking also relates to less frequent negative affect among teachers, such as feeling distressed, upset, guilty, nervous, and ashamed. Interestingly, perceived support within the external environment played a stronger role than the internal creative self-efficacy teachers held. Effects of this environmental support were substantial in size. Organizational learning research in schools focused on creativity (Rubenstein et al., 2013; Anderson et al., 2019) can go further to understand why these perceptions are related so strongly to aspects of teacher well-being.

The qualitative analyses about teachers’ innovations revealed that metaphor can play a role in how teachers and students conceptualize the challenging and abstract circumstances of a COVID-19 pandemic. Metaphors can be a powerful tool for sensemaking and motivation. For instance, using the metaphor of an educational pathway as a “journey” has been shown to have a positive effect on students’ academic intentions and effort (Landau et al., 2014). The Foundation Course modeled the use of a “river journey” to describe the teachers’ own experience in creative development, which seeded teacher innovations, such as having students create a “Metaphorest” in class, gathering metaphor for content they were learning. The Foundation Course focused on metaphor as an important creative learning tool, inherent in the way we think and make sense of the world across cultures and contexts. The role of metaphor in creative teaching and learning, especially in making sense of and managing the stress of crises and uncertainty, is a promising area for research in bridging creativity and education.

## Limitations

The quantitative survey data were correlational and cross-sectional with a relatively small sample, so analyses are limited in their scope and relationships are largely exploratory. It is not possible to distinguish directionality in the potential influence between creativity and well-being factors within this study. Given that the survey used self-report measures with Likert or frequency scales, results may face issues of monomethod bias, though the lack of statistically significant correlations between many factors makes this possibility appear less of a threat. Teachers self-selected into this study by voluntarily signing up for the Foundation Course, indicating they likely had a pre-existing interest in creativity. Teachers were also predominantly from rural areas, indicating that some aspects of their experience may not generalize to a more urban context. This study was limited in the range of creative resources included and did not incorporate cognitive and behavioral aspects of creativity. Future studies on this topic would be enhanced with additional dimensions of creative potential, such as divergent thinking originality.

## Conclusion

Teacher beliefs, affect, and attitude toward creativity and their sense of environmental support for student creativity in their school play a role in their emotional well-being, their secondary traumatic stress, their buoyancy, and their state of joy in teaching. However, teachers’ freedom and encouragement to be creative in their work has not been prioritized in education (Glaveanu and Beghetto, 2017), and that may come at a cost to their well-being in their work and their resilience in the face of crises, such as the COIVD-19 pandemic. Distance learning through the pandemic added stress to teachers’ lives and negatively affected the capacity of most teachers to provide creative learning opportunities. Still, a quarter of teacher participants recognized that the conditions of the pandemic energized their own creativity. Moreover, when teachers were supported with training and materials to innovate and adapt creative learning routines to distance learning, most teachers generated ideas aimed at supporting students’ creative and social-emotional development, simultaneously. This study adds to the growing research base describing how creative development of teachers and school support for creativity nurtures teacher longevity and engagement in this challenging but rewarding and consequential profession.

## Data Availability Statement

The raw data supporting the conclusions of this article will be made available by the authors, without undue reservation.

## Ethics Statement

The studies involving human participants were reviewed and approved by IntegReview. The patients/participants provided their written informed consent to participate in this study.

## Author Contributions

RA contributed to the conceptual development and framing of the study. All authors contributed to the refinement of measures included, data analysis, reporting of results, and revisions to the drafts of the study.

## Conflict of Interest

The authors declare that the research was conducted in the absence of any commercial or financial relationships that could be construed as a potential conflict of interest.
