# Supplementary material for: Generating Buoyancy in a Sea of Uncertainty: Teachers Creativity and Well-Being During the COVID-19 Pandemic
Source: Front Psychol. 2021 Jan 18;11:614774. doi: 10.3389/fpsyg.2020.614774 (PMC7848228; doi:10.3389/fpsyg.2020.614774)
Supplement: Supplementary file 1 [file Data_Sheet_1.pdf]

Appendix

Sample Creative Avatars and Description

Example #1

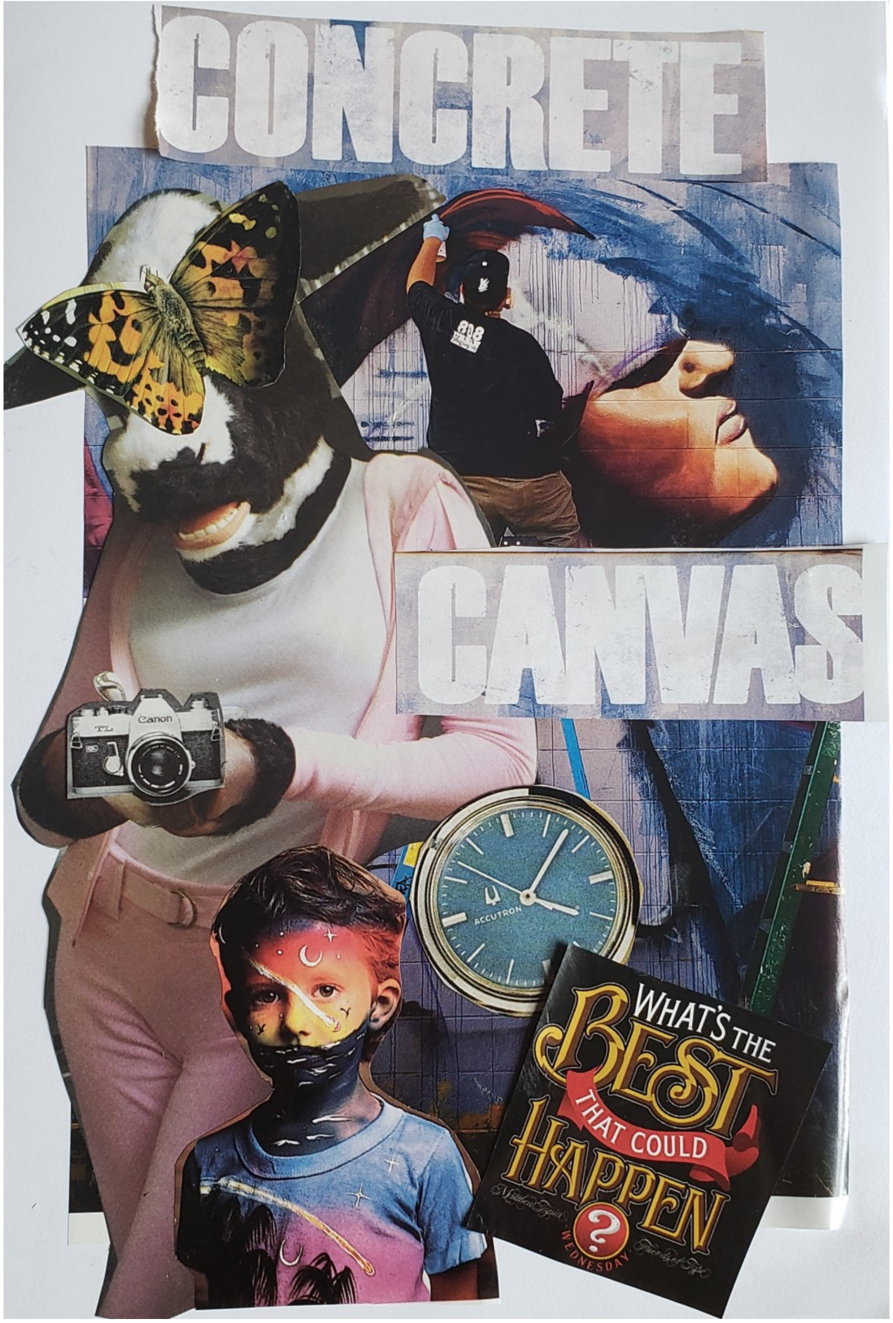

My avatar represents that anything can be a resource depending on how you look at it. A wall can be a concrete canvas a face can be a canvas. If you take the time to look at the world around you, you will find resources for creativity in just about anything. The butterfly represents the a transformed vision. I have really had to rethink, re-imagine and transform my curriculum using all of the resources that I can find in the world around me to teach art online during a pandemic.

Example #2

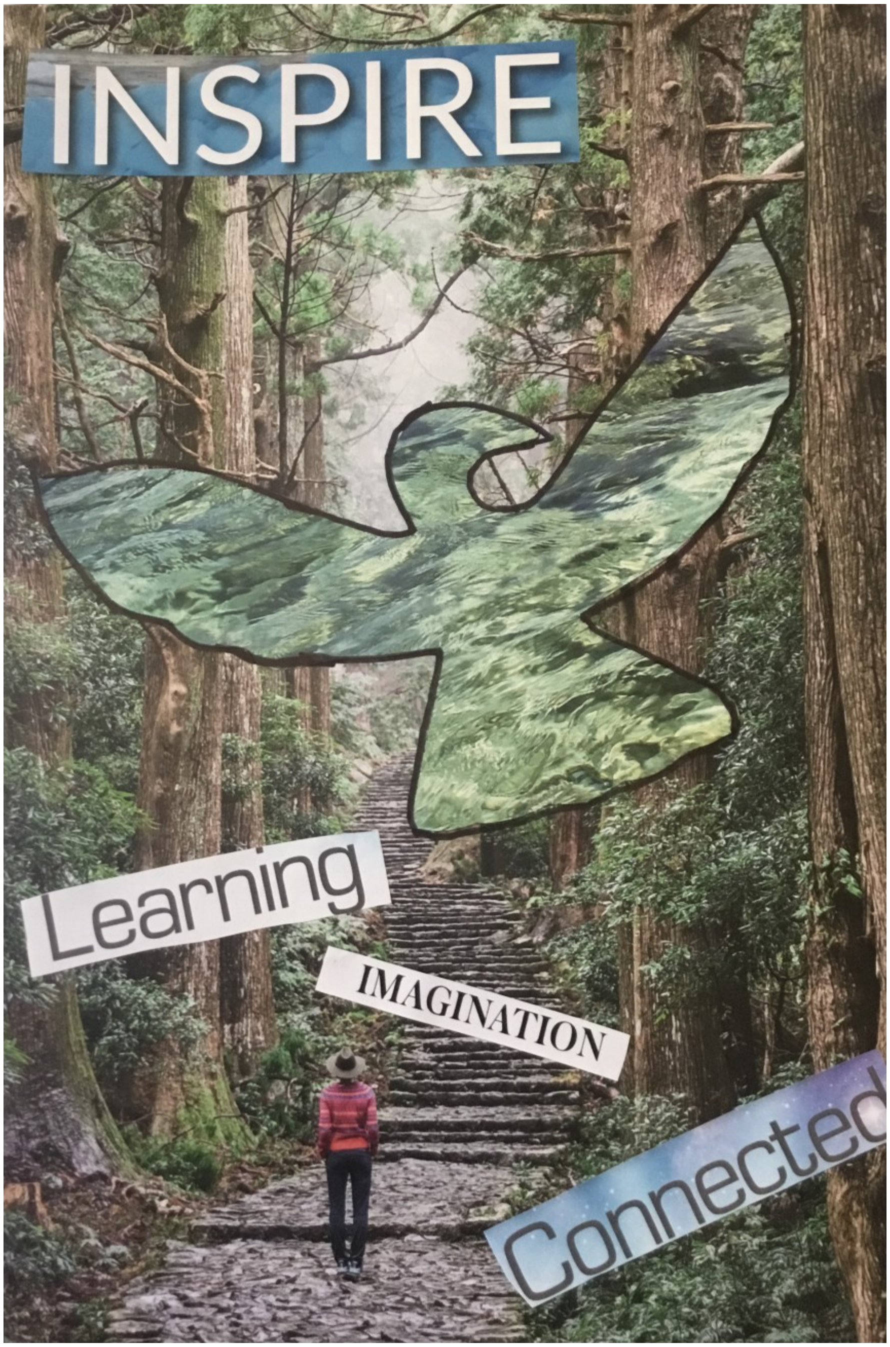

As I was browsing through my stack of old magazines to start this activity, I found myself gravitating towards images of nature. The background is the wildness of trees, the expanse of nature that dwarfs the human concerns. The raven - free, clever, curious - is cut out of an image of clear ocean water, representing clarity of thought and emotion. I added some power words as well because I am a very verbal person and often think in words instead of pictures. These ideas along with the beauty and power of the natural world are what sustain me and keep me healthy enough to partake in the creative process.

Example #3

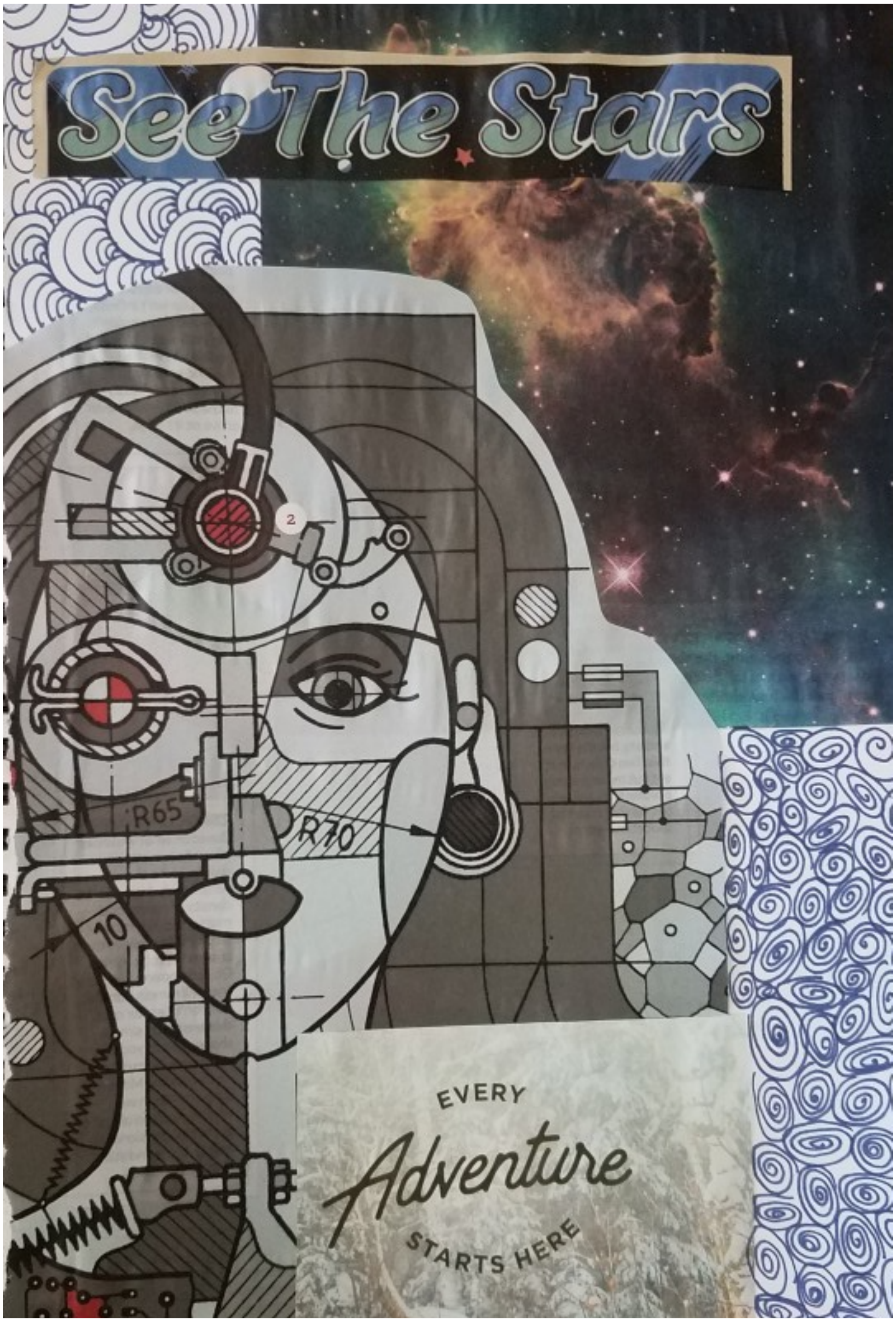

My avatar focuses on adventure and embracing the unknown. I look to the world around me as a tool and use natural resources to help inspire my own creativity.

Example #4

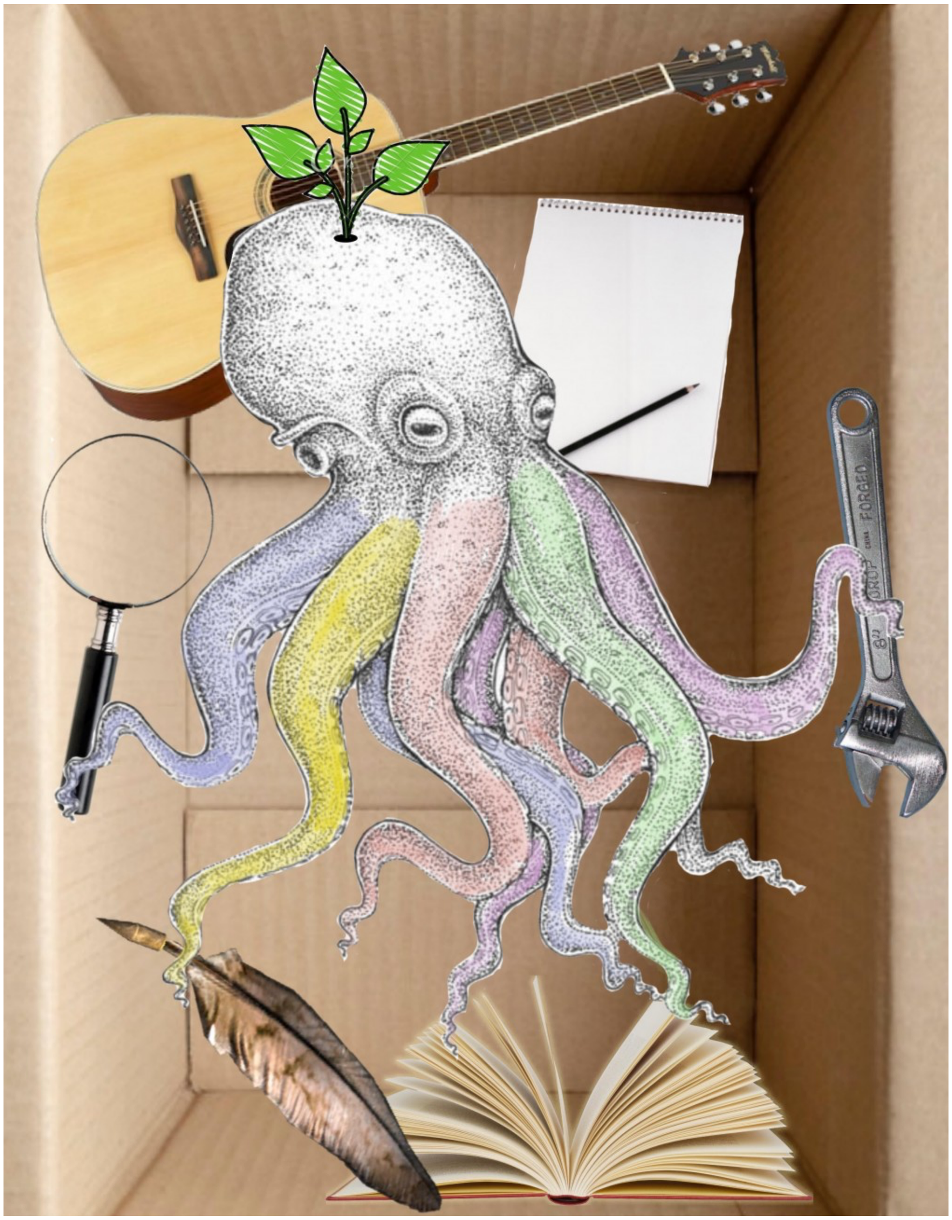

Mine came out weird, for sure. I didn't have the resources available so I did mine digitally using images I found online. The octopus came from my feeling that I've always got my hands busy with too many projects and ideas that I never really focus on one thoroughly. Many of the images are just representative of the kinds of things I like to create (books, sketches, music, etc) but there are a few that are more symbolic. The wrench is there because I feel like one of my strengths (especially in teaching) is the ability to tweak someone else's ideas or resources to work for me. The magnifying glass is there to symbolize my perfectionist nature that often gets in the way of sharing my work with others. There's a plant growing out of its head to show my focus on growth mindset, and all of it is contained in a box because I am sometimes held back by the boundaries I feel are placed around me, whether real or imagined.

Scales and Items Used in the Quantitative Analysis

Likert Scale
1 = Strongly Disagree
2 = Disagree
3 = Slightly Disagree
4 = Slightly Agree
5 = Agree
6 = Strongly Agree]

Creative Self-Efficacy in Teaching

1. I feel that I am good at coming up with novel ideas for teaching.
2. I have confidence in my ability to solve problems creatively.
3. I have a knack for helping students to elaborate on their own unique ideas
4. I am capable of teaching my students to find connections in seemingly unconnected ideas.
5. I am confident in my ability to develop a classroom atmosphere that welcomes creative risk-taking.
6. I can help many students become more creative.

Self-Theory Growth Creative Mindset

1. As a teacher, I can always increase my creative potential through learning and practice.
2. No matter how much creative potential I have as a teacher, I can always develop further.
3. I can change even my basic level of creative skills.
4. I can always change or increase my creativity in my instruction.

Self-Theory Fixed Creative Mindset

1. I can learn how to do and make new things, but I can't really change my basic level of creative potential.
2. I have a certain amount of creative potential, and I can't really do much to change it.
3. To be honest, I can't really change how creative I am.
4. My creative potential is something about me that I can't change very much.

Environmental Support and Encouragement for Creativity

1. My current school environment places little value on the development of student creativity.
2. It is a priority in my school to increase students' inventiveness.
3. My current school environment does not encourage teachers to produce independent thinkers.
4. My administration encourages me to foster innovative thinking in my students.
5. My school's priorities do not include teaching students to think creatively.

Need for Closure: Ambiguity in Teaching

1. When teaching, I don't like situations that are uncertain.

2. I feel uncomfortable when I don't understand why my instructional routine or lesson doesn't go the way I expected.
3. I dislike it when a student's question or statement could mean many different things.
4. I feel discomfort when a student asks me a question I don't know the answer to.
5. I don't like when a student's idea or question distracts the class from what I am trying to teach.

Buoyancy in Teaching

1. I don't let teaching stress get on top of me.
2. I'm good at bouncing back from a rough teaching day at school.
3. I think I'm good at dealing with teaching pressures.
4. I don't let a rough day of teaching affect my confidence.
5. I'm good at dealing with setbacks in my teaching job (e.g., students struggling with my teaching, negative feedback from others)

Dispositional Joy Scale

1. As a teacher, I often feel bursts of joy.
2. As a teacher, I can find joy in almost any occasion.
3. I would say that most of the occasions in my life as a teacher bring me joy.
4. Many things about being a teacher bring me delight.
5. I would say that I am an enthusiastic teacher
6. Good things happen to me all the time as a teacher.
7. Even when things aren't going well in my teaching, I can still feel joy.
8. Even in the midst of bad situations in teaching, I can usually find something to rejoice about.
9. As a teacher, I consistently feel a subtle but enduring feeling of joy.

Creative Anxiety

In this section, you will be presented with various situations and experiences that may cause tension, apprehension, or anxiety. For each situation, please choose the response that best describes how much it would make you feel anxious.

Response options:
1 = Very Slightly or not at all
2 = A little
3 = Moderately
4 = Quite a bit
5 = Extremely

1. Having to come up with a unique way of doing something.
2. Having to think in an open-ended and creative way.
3. Having to think "outside of the box"
4. Focusing on novelty over precision when doing something.

Positive and Negative Affect Scale

The following scale consists of a number of words that describe different feelings and emotions. Read each item and then mark the appropriate answer in the space next to the word. Indicate to what extent you have felt this way generally in your work as a teacher this past year.

Response options:
1 = Very Slightly or not at all
2 = A little
3 = Moderately
4 = Quite a bit
5 = Extremely

____Interested
____Distressed
____Excited
____Upset
____Guilty
____Enthusiastic
____Proud
____Irritable
____Ashamed
____Inspired
____Nervous
____Determined
____Attentive
____Jittery
____Active
____Afraid

Secondary Traumatic Stress Scale

Intrusion Subscale (add items 2, 3, 6, 10, 13),
Avoidance Subscale (add items 1, 5, 7, 9, 12, 14, 17),
Arousal Subscale (add items 4, 8, 11, 15, 16),
TOTAL (add Intrusion, Arousal, and Avoidance Scores)

Read each statement then indicate how frequently the statement was true for you during the last term of school by circling the corresponding number next to the statement.
1 = Never
2 = Rarely
3 = Occasionally
4 = Often
5 = Very often

1) I felt emotionally numb
2) My heart started pounding when I thought about my work with students

3) It seemed as if I was reliving the trauma(s) experienced by my students
4) I had trouble sleeping
5) I felt discouraged about the future
6) Reminders of my work with students upset me
7) I had little interest in being around others
8) I felt jumpy
9) I was less active than usual
10) I thought about my work with students when I didn't intend to
11) I had trouble concentrating
12) I avoided people, places, or things that reminded me of my work with students
13) I had disturbing dreams about my work with students
14) I wanted to avoid working with some students
15) I was easily annoyed
16) I expected something bad to happen
17) I noticed gaps in my memory about working with some students
